# Investigation of constitutive properties of high plasticity clay soils mixed with crushed rubber tire waste

**DOI:** 10.1016/j.heliyon.2024.e26655

**Published:** 2024-02-17

**Authors:** Abolfazl Eslami, Davood Akbarimehr, Alireza Rahai, Moses Karakouzian

**Affiliations:** a Department of Civil and Environmental Engineering, Amirkabir University of Technology, Tehran, Iran; b Department of Civil Engineering, University of Nevada, Las Vegas, United States

**Keywords:** Failure analysis, High plasticity clay soil (HPCS), Crushed rubber tire waste (CRTW), UCS, Rubber waste shape, SEM and binocular analysis

## Abstract

Addressing the enormous waste resulting from discarded worn rubber tires is an environmental challenge. Recycling and using crushed rubber tire waste (CRTW) in construction materials can help in addressing this challenge. This study investigates the effect of addition of CRTW on the engineering properties of high plasticity clay soils (HPCS). There is a paucity of research in the application of CRTW in HPCS. This research tries to fill this research gap. Specifically, this study seeks to investigate the effect of mixing CRTW on the constitutive properties of HPCS. After identifying a locally available HPCS, mixtures of the clay and several percentages (0%, 6%, 12%, 18%, and 24%) by weight of CRTW were prepared. A range of CRTW shapes and sizes were investigated. Three different particle shapes of CRTW (granular rubber, rubber chips, and rubber fiber), and two particle sizes (fine and coarse) were studied. The parameters studied included unconfined compressive strength (UCS), strain at failure, post-peak strength loss (PPSL), modulus of elasticity, failure modes/mechanisms, repeatability of tests results, and examination of CRTW particles and mixtures via binocular and SEM microscope. Our findings unveiled that the highest level of repeatability was observed in granular CRTW, with a maximum variability of only 5%. Moreover, the mixtures containing granular CRTW exhibited, on average, 10% and 15% higher strength and modulus of elasticity, respectively, in comparison to mixtures incorporating other shapes of CRTW. In general, the HPCS-CRTW mixtures displayed higher shear strains, averaging 25% greater than pure HPCS. Furthermore, the addition of CRTW to HPCS resulted in a reduction of its PPSL and a transition in behavior from brittle to slightly ductile. Examination of failed specimens revealed the existence of two primary failure modes: shear plane failure and shear plane failure accompanied by multiple vertical cracks within the mixtures. These results suggest that the utilization of granular CRTW in HPCS can improve certain properties of HPCS. However, it is advisable to limit the rubber content in this mixture to 6% to mitigate significant adverse effects on its strength.

## 1 Introduction

One type of waste that demands attention is the leftover rubber from worn tires, commonly referred to as rubber waste (RW). Each year, the global tire production increases, consequently leading to a surge in the volume of RW that requires effective management. This underscores the critical importance of making informed decisions regarding RW waste management. In light of engineering and environmental considerations, the most prudent approach for mitigating its adverse environmental impact is to incorporate RW into industrial processes [[1], [2], [3]].

Research has demonstrated that RW can be effectively utilized as a lightweight filler, enhancing the strength properties of various civil engineering applications [[4], [5], [6], [7]]. Given the potential advantages of employing RW mixtures in construction materials, numerous studies have been conducted on this subject [[8], [9], [10], [11], [12]]. The use of the mixture of soil and rubber is also seen in a number of researches [[13], [14], [15], [16]].

Furthermore, the integration of rubber with concrete has been explored in engineering practice, yielding valuable insights into increasing the utilization of waste rubber. Some researchers have successfully combined bentonite with RW to produce concrete with favorable strength properties [17]. The pre-treatment of rubber for use in concrete is of considerable significance, and a cost-effective, time-saving method has been proposed in another study [18].

In-depth analyses of crumb rubber concrete characteristics have been carried out using analytical modeling, with results indicating the feasibility of empirical models for estimating CRC properties [19]. Additionally, studies have investigated the use of Rubcrete in hybrid double-skin tubular columns, demonstrating enhanced hoop and axial strain capacities, particularly with fine rubber particles [20].

Research has also delved into the compressive behavior of FRP-confined expansive rubberized concrete, highlighting the potential for improving strength properties by harnessing the incompressibility of rubber [21]. Experimental studies on the push-off and pull-out bond behavior of CRC composite slabs have generated valuable data for researchers in the concrete field [22]. Additionally, research has explored composite walls comprising foam rubberized concrete and profiled steel skin subjected to eccentric compressions, offering practical equations for predicting interaction diagrams for PSCW structures [23].

Various studies have examined the effect of temperature on rubber concrete. For instance, research has investigated the influence of multi-scale fibers on the residual compressive properties of rubberized concrete exposed to elevated temperatures, revealing a relatively ductile fracture after exposure to 800 °C [24]. Another study explored the uniaxial tensile properties of multi-scale fiber reinforced rubberized concrete after exposure to elevated temperatures, identifying the loss of strain-hardening capability when temperatures reached 400 °C [25].

Furthermore, rubber has been used in various ways to enhance material properties or investigate its effects on materials [[26], [27], [28], [29], [30], [31], [32], [33], [34], [35]]. Additionally, researchers have evaluated the use of a combination of rubber waste and other materials in construction materials [[36], [37], [38], [39], [40], [41], [42], [43]].

Researchers have also harnessed this material to improve soil properties [[44], [45], [46], [47], [48], [49], [50]]. Geotechnical studies involving soil-rubber mixtures have indicated the suitability of this combination for various geotechnical engineering applications, including machine foundations and retaining walls [6].

Research on the mixture of RW with low-plasticity clay soils has demonstrated that the strength of the mixture may fluctuate depending on the RW content, but its density consistently decreases as RW content increases [29,51]. Tests conducted by other researchers have also revealed an increase in shear strain and a decrease in strength in soil-RW mixtures with increasing RW content [52]. One study reported that the addition of RW to the soil can lead to either an increase or decrease in strength, with a potential 40% increase in UCS achievable by adding 5% RW to the soil [43]. In a separate study, the use of 10% RW in low-plasticity clays was shown to increase its strength by approximately 45% [53]. However, it is important to note that while some studies have observed strength improvements with RW, several others have reported the opposite effect. For example, one research found that adding 6% RW to clay reduced its strength by about 40% [54]. Jastrzębska (2019) found that both soil type and RW type significantly influenced mixture strength [55]. In a study by Soltani et al. (2020) on HPCS and RW mixtures, results indicated an enhancement in geotechnical properties with the addition of RW. This study used up to 20% rubber content in combination with soil, utilizing one type of crushed rubber [56].

Furthermore, the dynamic behavior of low-plasticity clay soil mixtures with RW has been explored by multiple researchers [57,58]. Another study by Soltani et al. (2021) on HPCS and RW mixtures demonstrated that the addition of Sodium Alginate Biopolymer could further enhance the geotechnical properties of the mixture. In this research, rubber content of up to 10% and one type of crushed rubber were employed. According to this study, the longer the Sodium Alginate curing duration and/or the higher the additive content, the greater the UCS [59]. Table 1 provides a list of recent studies on clay-RW mixtures.

**Table 1:** Recent clay and waste rubber mixtures studies.

Soil type	Type of waste	Content (%)	Size (mm)	Research Topic	References
CL	RW powder	0–30%	0.1–1	Geotechnical Characteristics	[12]
CL	fibers	0–10%	2–3	strength characteristics	[60]
Red clay	Granular	5–25%	0.1–1 & 1-5	Strength characteristics	[55]
CL	Granular	0–30%	<1 & 1-5	Densification Characteristics	[11]
CL	Granular	0–30%	Different size	Geotechnical properties	[27]
CH	TDA	5–20%	Different size	Soil improvement	[56]
Red clay & Kaolin	Granular	5–25%	0.1–1 & 1-5	Strength Characteristics	[58]
Tehran clay	Granular	0–30 %	<1 & 1-5	Dynamic Characteristics	[57]
CH	TDA	0–10%	0.075–2	Soil improvement	[59]
Different	Different	Different	Different size	Compaction Characteristics	[61]
CL	4 types of rubbers	0–30%	Different size	Failure analysis	[7]
CL	Granular	0–30%	Different size	Elasto plastic analysis	[6]
CL	RW powder	0–100%	0.1 to 1	Deformation analysis	[3]

The classification of fine-grained soils is typically determined using the Casagrande chart [49]. Among fine-grained soils, those with high plasticity exhibit a liquid limit (LL) exceeding 50 and a plasticity index (PI) surpassing 20. Given the prevalence of such soils in various regions and their significant role in engineering applications, it becomes imperative to investigate the characteristics of High Plasticity Clay Loam (HPCL) when blended with CRTW.

While some studies have explored the properties of clay-RW mixtures, there has been a notable scarcity of research dedicated to HPCL-CRTW mixtures. Furthermore, existing studies have typically focused on a single type of rubber and limited rubber content, employing various methodologies. Notably, the influence of CRTW shape on HPCL-CRTW mixtures' stress/strain behavior and Failure Envelopes parameters has remained largely unexplored, rendering this research novel and practically relevant in the context of incorporating CRTW in engineering practices.

Moreover, this study introduces a new mold with greater diameter and height for the preparation of uniaxial samples, thereby enhancing result accuracy. By investigating the impact of both the quantity and shape of rubber particles on soil-rubber mixture properties, this research aids in optimizing these parameters for cost-effective project outcomes. Additionally, the examination of failure mechanisms within soil-rubber mixtures enhances our understanding of their behavior.

It is crucial to replicate these investigations across diverse soil types to ensure a comprehensive understanding applicable to a wide array of projects. The strength of this mixture is contingent on several factors, including soil type, rubber type, and the quantity and shape of rubber particles [46,47].

In line with the study's objectives, this research delves into the geotechnical properties of HPCL-CRTW mixtures, encompassing Unconfined Compressive Strength (UCS), failure strains, post-peak strength, modulus of elasticity, repeatability, and failure modes/mechanisms. This comprehensive examination involves a series of geotechnical experiments and UCS tests to scrutinize how the mixture's behavior is influenced by the quantity, shape, and size of rubber waste particles. Additionally, SEM and binocular microscopy were employed to analyze the mixture's structure, while the results of X-ray diffraction (XRD) tests aided in further characterizing the soil.

## 2 Materials and methods

### 2.1 Materials

• **Soil**

**Fig. 1:**
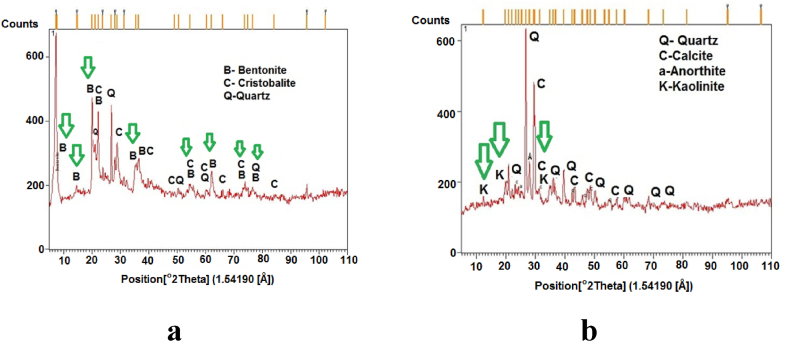
XRD analysis of the soils used for research. (a) Bentonite. (b) Tehran clay.

The primary objective of this study was to investigate the behavior of a High Plasticity Clay Soil (HPCS) and Crushed Rubber Tire Waste (CRTW) mixture. To create the soil utilized in our experiments, a blend of 60% Tehran clay [62] and 40% bentonite was employed. X-ray Diffraction (XRD) testing was conducted on samples of both Tehran clay and bentonite, as it plays a pivotal role in identifying clay minerals. The results of the XRD test are depicted in Fig. 1a, b.

**Fig. 2:**
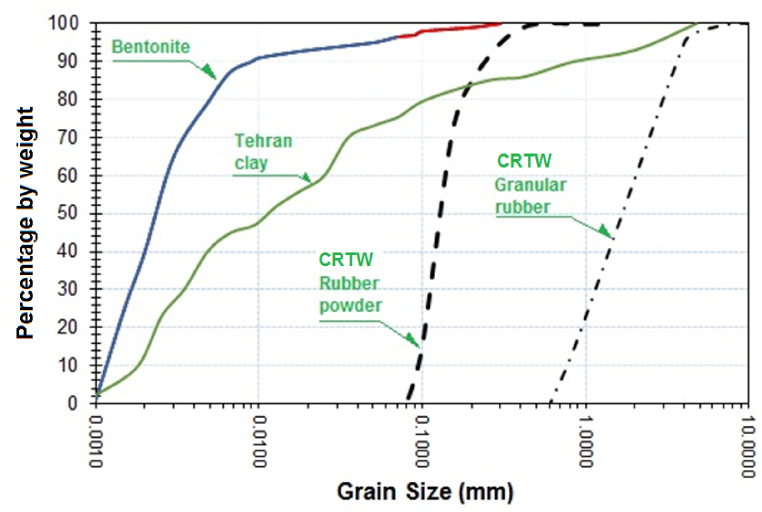
Gradation curve of clay soils, rubber powder and granular waste rubber.

Soil gradation diagrams provide valuable insights into soil grain size characteristics. Fig. 2 illustrates the gradation diagram of Tehran clay and bentonite, the constituent soils used in our experiments. To further enhance soil characterization, hydrometric tests were performed. The hydrometric test for determining the particle size of the fine fraction of the soil adhered to the procedures outlined in ASTM-D7928. Additional tests were conducted in accordance with ASTM-D698, ASTM-D4318, ASTM-D854, and ASTM-D698 to determine moisture content, Atterberg limits, specific mass, maximum dry unit weight, and optimum moisture content, respectively.

Based on the outcomes of these experiments, the soil exhibited a liquid limit of 75 and a plasticity index of 35. According to the Unified Soil Classification System (USCS) and ASTM-D2487, the soil falls into the category of HPCS-CH type clay. The specific mass of this clay is 2.7, with a maximum dry unit weight of 14.9 kN/m³ and an optimum moisture content of 21%.

• **CRTW**

The rubber utilized in this study was derived from discarded tires, constituting rubber waste sourced from these worn-out tires. The specific gravity of this rubber was measured at 1.11. A comprehensive chemical analysis revealed the rubber's composition, comprising sulfur (S, 1.2%), zinc (Zn, 1.6%), oxygen (O, 9.3%), carbon (C, 87.39%), aluminum (Al, 0.14%), magnesium (Mg, 0.23%), and silicon (Si, 0.14%).

**Fig. 3:**
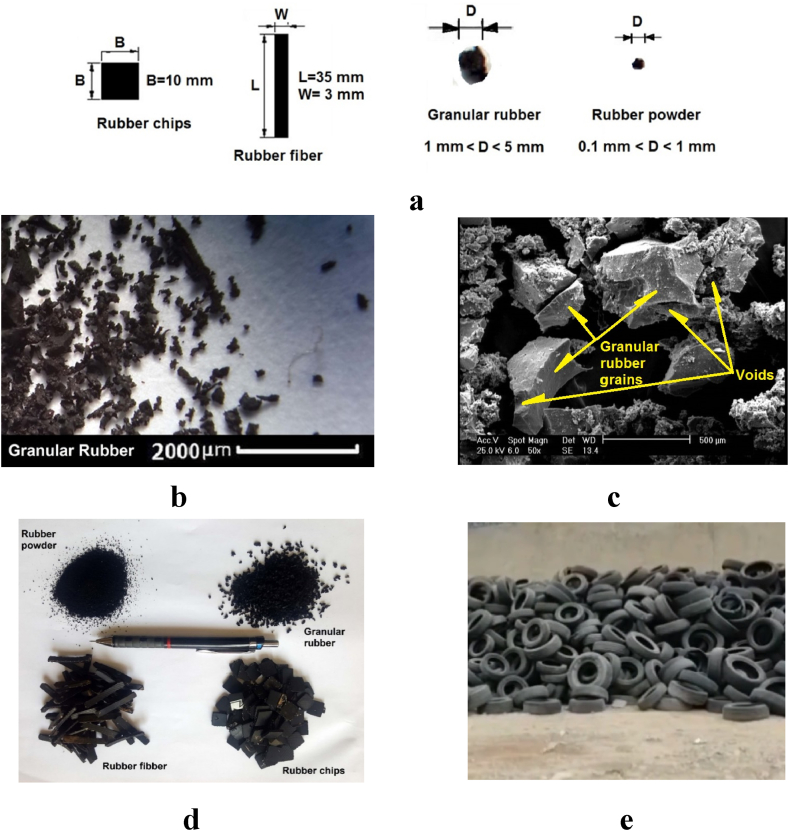
Waste rubbers used in this study. (a) Shape and size of rubbers. (b) Binocular view of granular rubber at 10× magnification. (c) SEM view of Granular rubber at 50X. (d) Picture of CRTWs. (e) Waste tires.

Recognizing the pivotal role of rubber particle shape in influencing the behavior of soil-rubber mixtures, our experiments incorporated rubber specimens of three distinct shapes: granular, chips, and fiber. Images showcasing these rubber waste varieties are depicted in Fig. 3a. In light of the prevalent availability of granular rubber waste in the market, we opted to employ granular rubber particles of two different sizes, hereafter referred to as granular and powder. Furthermore, acknowledging the significance of the rubber quantity in the mixture, we conducted experiments with five varying rubber proportions: 0%, 6%, 12%, 18%, and 24% of the soil. Fig. 3b and c provide magnified views of the used rubber powder, captured through both a binocular microscope and an SEM microscope. These images reveal that rubber particles exhibit non-spherical, irregular three-dimensional shapes with a length-to-width ratio exceeding 1, along with voids present on their surfaces. Fig. 3d displays an image of the rubber waste employed in our research, while Fig. 3e offers a glimpse of the location from which the rubber waste was collected. This location underscores the substantial volumes of waste generated and its potential environmental impact.

For this investigation, rubbers were obtained through the mechanical crushing of worn tires, as illustrated in Fig. 3b.

### 2.2 Test, test procedures and Test Matrix

• **Uniaxial Strength Tests (UCS)**

To measure the strength of HPCS-RW mixtures, uniaxial strength tests (UCS) were performed according to ASTM D2166. The uniaxial strength test is used when the confining pressure is absent or very limited. The stress-strain curves obtained from the results were used to determine various parameters including modulus of elasticity, UCS, axial strains at the moment of failure, and PPSL among others. This test was performed on cylindrical specimens at a rate of 1 mm/min.

• **HPCS -CRTW Mixtures and Tests Matrix**

One of the pivotal aspects of this research centered on the amalgamation of CRTW with High Plasticity Clay Soil (HPCS). To ensure the practicality of our findings, all specimens were meticulously crafted by adhering to the maximum dry unit weight and optimum moisture content standards. The mixture specimens were meticulously prepared by incorporating varying percentages of CRTW into the soil: 0%, 6%, 12%, 18%, and 24%. These particular percentages were selected based on recommendations from prior studies in this field [49]. It's worth noting that the upper limit for rubber content was capped at 24% since augmenting the rubber content beyond this range (>24%) substantially alters the mixture's behavior.

**Fig. 4:**
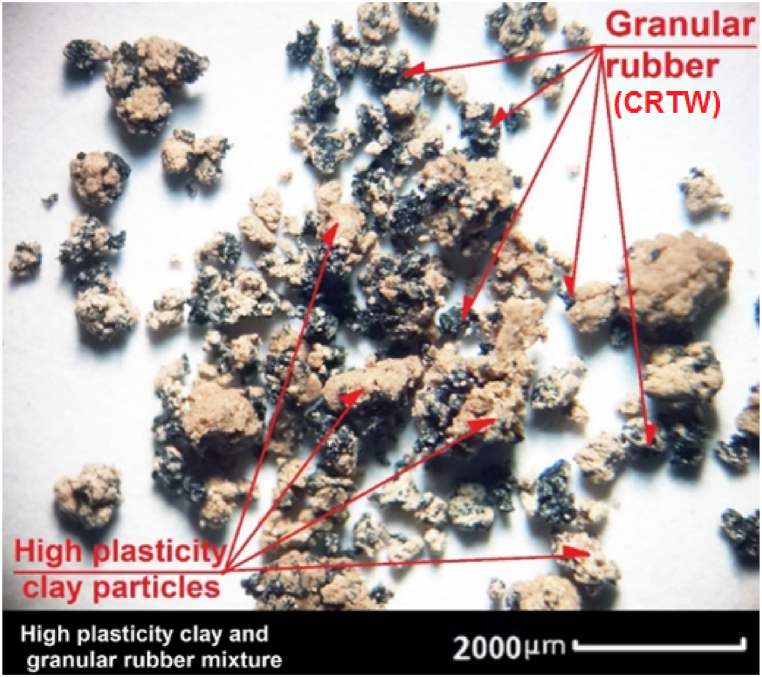
Binocular view of soil and granular rubber at 20× magnification.

Table 2 provides an overview of the composition of different HPCS-CRTW mixtures employed in our experiments. Each specimen's nomenclature comprises the soil content percentage of the mixture, followed by the type and quantity of rubber incorporated into the blend. For instance, the specimen denoted as HPCS88G12 represents a mixture consisting of 88% HPCS and 12% granular rubber. Fig. 4 offers an enlarged image of the soil-rubber mixture, showcasing the strong cohesion evident within the blend.

**Table 2:** HPCS-CRTW mixture proportions.

Sample code	Rubber shape	Composition	Sample code	Rubber shape	Composition
HPCS100RW0	No rubber	100% clay	HPCS 100RW0	No rubber	100% clay
HPCS 94G6	Granular	Clay+6% G	HPCS 94P6	Powder	Clay+6% P
HPCS 88G12	Granular	Clay+12% G	HPCS 88P12	Powder	Clay+12% P
HPCS 82G18	Granular	Clay+18% G	HPCS 82P18	Powder	Clay+18% P
HPCS 76G24	Granular	Clay+24% G	HPCS 76P24	Powder	Clay+24% P
HPCS 94C6	Chips	Clay+6% C	HPCS 94F6	Fiber	Clay+6% F
HPCS 88C12	Chips	Clay+12% C	HPCS 88F12	Fiber	Clay+12% F
HPCS 82C18	Chips	Clay+18% C	HPCS 82F18	Fiber	Clay+18% F
HPCS 76C24	Chips	Clay+24% C	HPCS 76F24	Fiber	Clay+24% F

**Note.**

*G: Granular rubber*.

*C: Chips rubber*.

*RW: Any type of rubber*.

*F: Rubber fiber*.

*P: Rubber powder*.

*HPCS: High plasticity clay soil*.

### 2.3 Specimen preparation

The meticulous preparation of suitable specimens for testing stands as a paramount aspect of this research endeavor. Given the size of rubber chips and fibers, it became imperative to employ a sizable mold for the creation of uniaxial specimens. The mold chosen needed to possess a minimum diameter that was at least 10 times larger than the diameter of the largest grain within the mixture. The specimen preparation method adopted here closely aligns with procedures employed in previous studies [49,57].

**Fig. 5:**
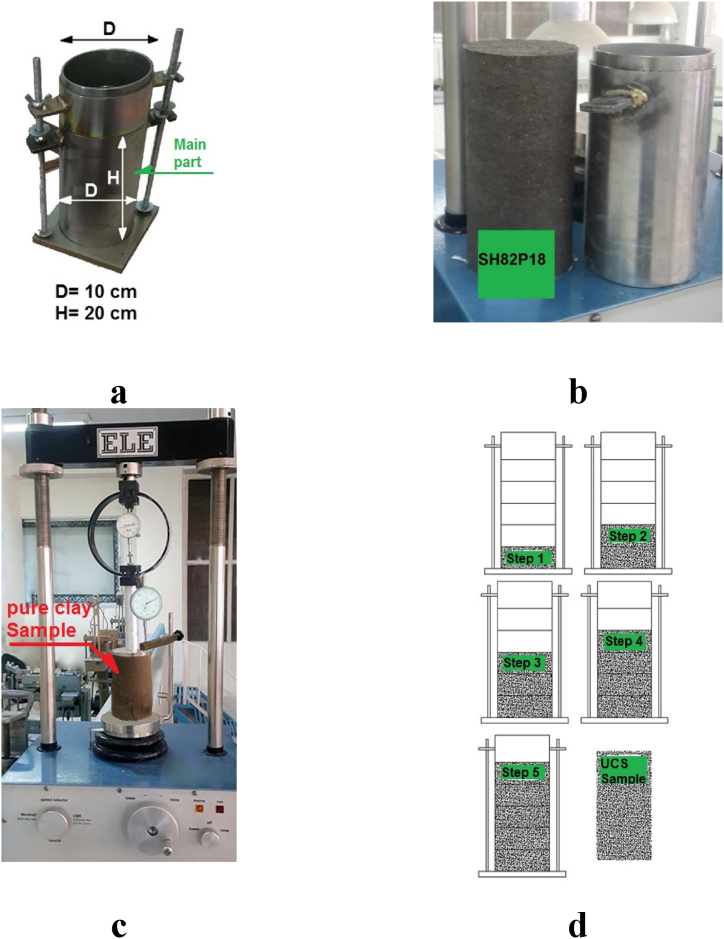
Details of UCS test. (a) Designed sampling mold. (b) Extrusion of sample HS_82_G_18_ from the mold. (c) UCS device and sample HPCS100RW0. (d) Sample preparation steps.

The utilized mold was capable of producing specimens with a height of 20 cm and a diameter of 10 cm, as depicted in Fig. 5a. This mold closely resembled the mold used for compressive testing, with the key difference being an adjusted height of the main part to 20 cm (in contrast to the 10 cm height in the compressive mold). Following the preparation of the mold and the application of suitable grease to its inner surface, the mixture was systematically deposited into the mold in five layers, each of which was compacted. For each layer, 20% of the total specimen weight was placed into the mold and compacted using a 5.5lb hammer until the layer's height was reduced to 4 cm. The height reduction was meticulously measured using calipers. To ensure uniformity and consistency in the specimen created from these layers, prior to depositing each new layer, the surface of the preceding layer underwent a scratching process with a spatula, resulting in the formation of vertical and horizontal grooves with a depth of 3 mm.

Upon the completion of the deposition of all five layers within the mold, the specimen was carefully extracted from the mold using a hydraulic jack. Fig. 5b provides an image of the HPCS82P18 specimen after its removal from the mold. Subsequently, the specimen was situated on a uniaxial strength test cap. Images illustrating the uniaxial strength testing machine, the specimen preparation process, and the final products stemming from this meticulous procedure are showcased in Fig. 5c and d.

## 3 Results and analysis of results

### 3.1 Tests repeatability

The repeatability of test results holds paramount importance in engineering applications, particularly in geotechnical engineering. In the context of this study, repeatability assumes a critical role as many projects necessitate precise predictions of mixture behavior and associated parameters. Hence, the higher the level of repeatability achieved in the results, the greater the accuracy in predicting mixture behavior. To assess repeatability, two specimens, identical in unit weight and moisture content, were crafted and tested for each combination of HPCS with granular rubber, rubber powder, rubber chips, and rubber fiber. All these specimens comprised 6% CRTW and 94% HPCS.

**Fig. 6:**
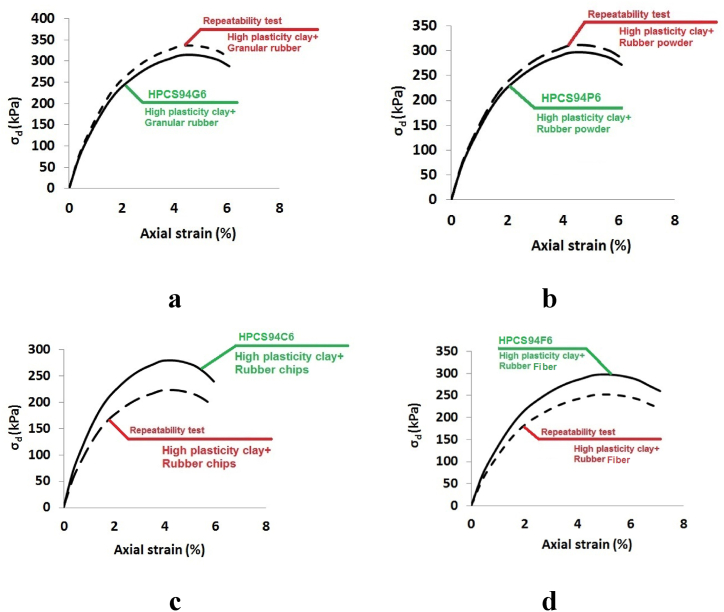
Evaluation of repeatability tests in clay and different shape of rubber mixtures. (a) Sample HPCS_94_G_6_. (b) Sample HPCS_94_P_6_. (c) Sample HPCS_94_C_6_. (d) Sample HPCS_94_F_6_.

The outcomes of these repeatability tests are depicted in Fig. 6(a-d). As illustrated by these results, among all the mixtures, the combination of HPCS with granular rubber and rubber powder exhibited the highest repeatability, reaching an impressive 95%. This exceptional repeatability can be attributed to the shape and size of rubber particles. Given the nearly homogeneous nature of granular mixtures, these results align with expectations. In contrast, due to the relatively lower homogeneity of specimens containing rubber fiber and rubber chips, their repeatability scores were slightly lower, at approximately 91%.

These findings suggest that mixtures of HPCS with granular CRTW yield more consistent and repeatable outcomes. Consequently, this enhances the feasibility of producing highly accurate predictions regarding their behavior, in contrast to mixtures involving CRTW chips or CRTW fibers. From a practical standpoint, it is strongly recommended to opt for granular CRTW when incorporating CRTW into HPCS mixtures.

### 3.2 Peak strength

**Fig. 7:**
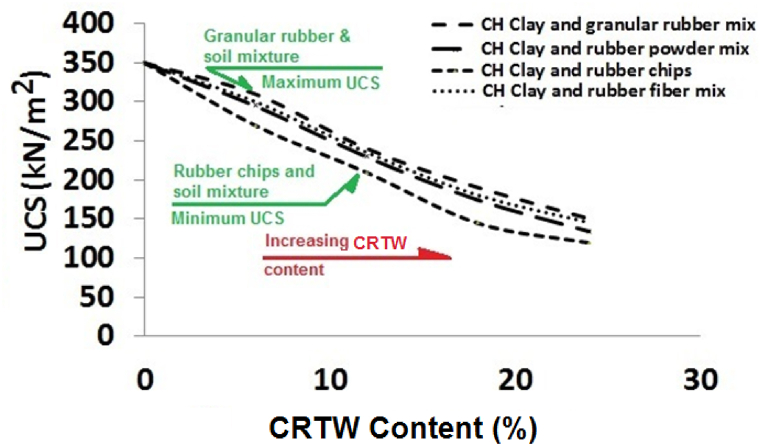
Effect of rubber content on UCS of HPCS-CRTW.

The peak strength of uniaxial specimens holds significant practical importance as it provides valuable insights into the suitability of different types of rubber within the mixture. In Fig. 7, we have plotted changes in the peak strength of these mixtures against their CRTW content. This figure reveals that the addition of 0%, 6%, 12%, 18%, and 24% granular rubber to HPCS resulted in a decrease in peak strength from 350 KPa to 310 KPa, 240 KPa, 190 KPa, and 150 KPa, respectively. Similar trends can be observed for the mixture of HPCS with rubber powder, where the addition of 0%, 6%, 12%, 18%, and 24% CRTW to HPCS led to peak strength reductions from 350 KPa to 295 KPa, 230 KPa, 175 KPa, and 135 KPa, respectively. These trends are also applicable to the mixtures containing rubber fiber, where the inclusion of 0%, 6%, 12%, 18%, and 24% CRTW in HPCS resulted in peak strength reductions from 350 KPa to 300 KPa, 235 KPa, 180 KPa, and 145 KPa, respectively. Notably, specimens containing rubber chips exhibited lower peak strengths compared to the other specimens. The introduction of 6%, 12%, 18%, and 24% of this type of CRTW led to peak strength reductions from 350 KPa to 270 KPa, 210 KPa, 145 KPa, and 120 KPa, respectively.

From these findings, it is evident that the mixture of HPCS with CRTW attains its highest peak strength when CRTW is in granular shape. The second and third highest peak strengths are associated with mixtures containing rubber fiber and rubber powder, respectively, while the lowest peak strength is observed in the mixture involving rubber chips. The superior strength exhibited by specimens containing granular rubber can be attributed to the non-spherical, irregular shape of these grains, which possess a length-to-width ratio exceeding one. This shape provides reinforcing properties that enhance the mixture's strength, akin to the reinforcement observed in cob (a traditional building material combining straw and clay mud) and steel-reinforced concrete. A similar but less effective reinforcing effect is evident in the mixtures of HPCS with rubber powder and rubber fiber, attributed to the smooth texture of rubber fiber (resulting from tire punching) and the smaller particle size of rubber powder compared to granular rubber.

The marked reduction in strength observed in the mixture of HPCS with rubber chips is primarily attributed to a significant decrease in HPCS cohesion, stemming from the substantial specific surface area of the added rubber chips.

Given the practical value of being able to predict the uniaxial strength of HPCS-CRTW mixtures based on the shape and quantity of CRTW, we formulated several equations for estimating the uniaxial strength of these mixtures. These equations, presented in Table 3, can greatly facilitate the practical utilization of CRTW in engineering projects. The strength estimation equations in Table 3 were derived from the data in Fig. 7 using linear regression, with Excel software utilized for this purpose. The equations, along with their associated R-squared (R^2^) values, were easily obtained using the "linear trendline tool" within the software.

**Table 3:** Evaluation of UCS in HPCS and CRTW mixtures.

Equation	Rubber shape	R square
UCS = −9.9 CRTW+351	Granular rubber	0.99
UCS = −9.7 CRTW +355	Rubber fiber	0.99
UCS = −10.5 CRTW +352	Rubber powder	0.97
UCS = −11.4 CRTW +349	Rubber Chips	0.93

### 3.3 Modulus of elasticity

**Fig. 8:**
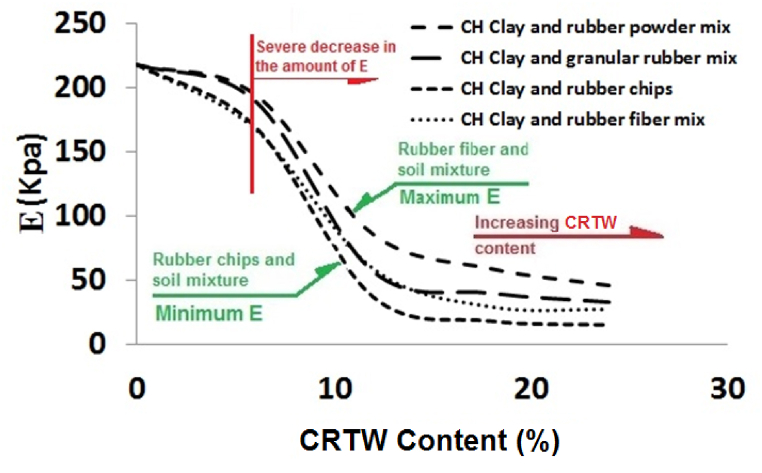
Effect of rubber content on modulus of elasticity of HPCS-CRTW mixture**s**.

The modulus of elasticity of soil is a critical parameter that engineers must carefully assess and consider in geotechnical engineering projects. This modulus represents the slope of the linear segment of the stress-strain curve and can thus be determined from the results of uniaxial strength tests. Fig. 8 presents the modulus of elasticity values derived from the stress-strain curves of various HPCS-CRTW mixtures. As illustrated in this figure, the inclusion of 6%, 12%, 18%, and 24% rubber powder led to changes in the soil's modulus of elasticity from 218 KPa to 195 KPa, 86 KPa, 59 KPa, and 46 KPa, respectively. Similarly, the inclusion of 6%, 12%, 18%, and 24% granular rubber reduced the soil's modulus of elasticity from 218 KPa to 190 KPa, 57 KPa, 40 KPa, and 33 KPa, respectively. In the case of rubber fiber, the corresponding changes were from 218 KPa to 168 KPa, 59 KPa, 29 KPa, and 27 KPa, respectively. For rubber chips, the changes ranged from 218 KPa to 170 KPa, 37 KPa, 18 KPa, and 15 KPa, respectively.

Collectively, the diagrams in this figure reveal that the addition of up to approximately 6% rubber has a minimal impact on the soil's modulus of elasticity, whereas higher rubber content leads to a sharp decrease in this modulus. However, adding more than 12% rubber has a comparatively limited impact on the modulus of elasticity. The relatively stable modulus of elasticity in the 12%–24% rubber content range can be attributed to the substantial amount of rubber present in the soil. It is worth noting that in this research, up to 24% rubber was added to the soil, and further increases in rubber content may result in more significant changes to the modulus of elasticity. Similar to the results of uniaxial strength tests, which demonstrated the influence of rubber content on mixture strength, these findings underscore the importance of selecting the appropriate amount of rubber to achieve the desired modulus of elasticity for the soil.

Comparing the modulus of elasticity of specimens containing different shapes of rubber reveals that specimens containing rubber powder exhibit the highest modulus of elasticity, while those containing granular rubber and rubber fiber have relatively lower values, with the lowest modulus of elasticity observed in the mixture containing rubber chips. The influence of material shape on the elastic modulus is well-documented. Due to variations in the contact surface of grains in different rubber waste shapes, the modulus of elasticity, like other parameters, is naturally affected by material shape. Similar observations regarding the effect of shape on the elastic modulus can be found in previous research, as exemplified by Vogl et al. (2021) [63].

This trend aligns with the findings of previous studies, including those by Akbarimehr et al. (2020) [49] and Akbarimehr and Fakharian (2021) [57], which reported a higher modulus of elasticity in mixtures of low-plasticity clay with rubber powder.

### 3.4 Failure analysis

#### 3.4.1 Stress-strain curves

**Fig. 9:**
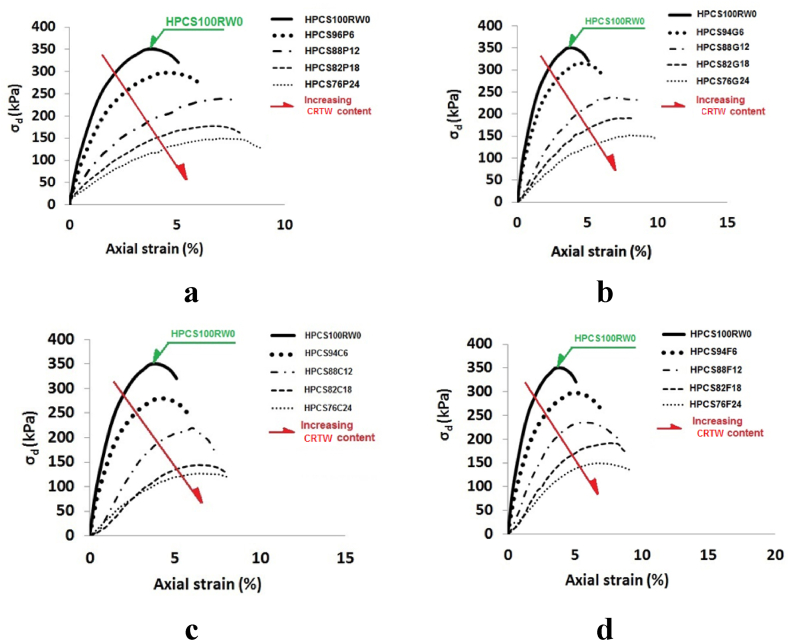
Stress-strain behavior in HPCS and CRTW mixtures. (a) HPCS soil and rubber powder mixture. (b) HPCS soil and granular rubber mixture. (c) HPCS soil and rubber chips mixture. (d) HPCS soil and rubber fibers mixture.

The stress-strain curves of HPCS-CRTW mixtures provide valuable insights into the performance of these mixtures. Fig. 9a – d illustrates the stress-strain curves of mixtures containing varying percentages of CRTW in different shapes. Across all mixtures, there is a noticeable decrease in strength to some extent as the rubber content increases. This trend is expected, considering that CRTW typically has lower strength compared to HPCS. However, the figure also reveals a noteworthy decrease in PPSL as the rubber content increases. While pure HPCS exhibits a sharp and sudden drop in PPSL, specimens containing CRTW show a more gradual decline, indicating a shift from brittle behavior to ductile behavior, which is quite intriguing. This behavior remains consistent regardless of the shape of CRTW, whether it is granular, chips, or fiber.

In general, specimens containing granular rubber and rubber powder exhibit lower post-peak strength than other prepared mixtures. The reduced PPSL in the CRTW-containing specimens can be attributed to higher axial strains at the point of failure and the reduced brittleness of these specimens. As the amount of rubber increases, the ductility of the mixture also increases, resulting in a lower PPSL. These axial strains will be discussed further in the following sections. These positive changes in PPSL represent significant outcomes of blending HPCS with CRTW, making this mixture a more favorable choice for practical applications.

#### 3.4.2 Failure strains

**Fig. 10:**
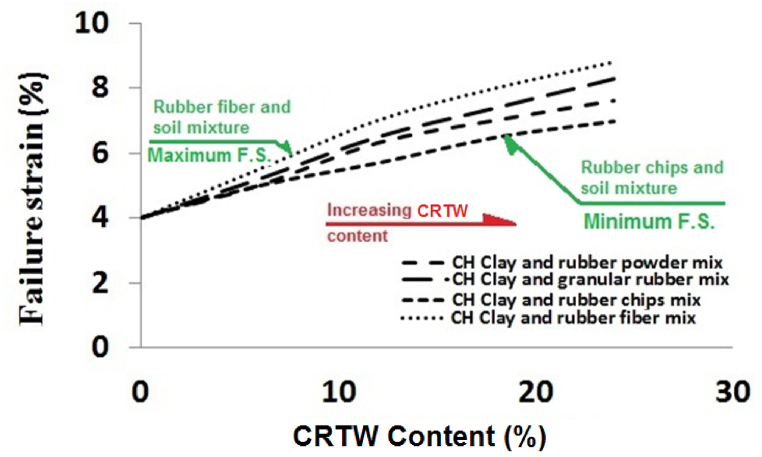
Effect of rubber content on failure strain of HPCS-CRTW.

Failure strains were extracted from the diagrams presented in Fig. 9a – d. Fig. 10 displays the variations in failure strain in relation to the rubber content for each rubber shape. In this figure, it is evident that incorporating 6, 12, 18, and 24% granular CRTW into HPCS resulted in increased failure strains, progressing from 4% for pure soil to 5.2%, 6.5%, 7.4%, and 8.3%, respectively. When rubber powder was introduced, the corresponding failure strains with 6, 12, 18, and 24% CRTW in HPCS were 5%, 6.3%, 7%, and 7.6%, respectively. The changes in failure strain for rubber fiber were similar to those of rubber powder and granular rubber, with failure strains increasing from 4% to 5.5%, 7%, 8%, and 8.8%, respectively. For specimens containing 6, 12, 18, and 24% rubber chips, the failure strains increased from 4% to 5%, 5.7%, 6.5%, and 7%, respectively.

Comparing the failure strains of specimens containing CRTW of different shapes clearly demonstrates that the shape of CRTW significantly influences the failure strains. The results reveal that the specimens with rubber fiber and granular rubber exhibit the highest failure strains, followed by those with rubber powder and rubber chips, in that order. The enhanced failure strains in specimens with granular rubber and rubber fiber can be attributed to their length-to-width ratio exceeding 1 and their reinforcing effect on the mixture. Fig. 3b provides a binocular microscope image of granular rubber, confirming the irregularity of these rubber granules and their high length-to-width ratio. The beneficial impact of granular rubber and rubber fiber on failure strains has also been observed in other studies, including Akbarimehr et al. (2020) [49]. The comparatively lower failure strain in specimens containing rubber chips is due to the reduced cohesion of the soil resulting from the addition of coarse rubber grains with a smooth surface, which also undermines the mixture's strength.

These findings indicate that as the quantity of CRTW added to HPCS increases, particularly in granular or fiber shape, the mixture's failure behavior transitions from brittle to ductile, contributing to improved performance in failure control.

#### 3.4.3 Types of failure

**Fig. 11:**
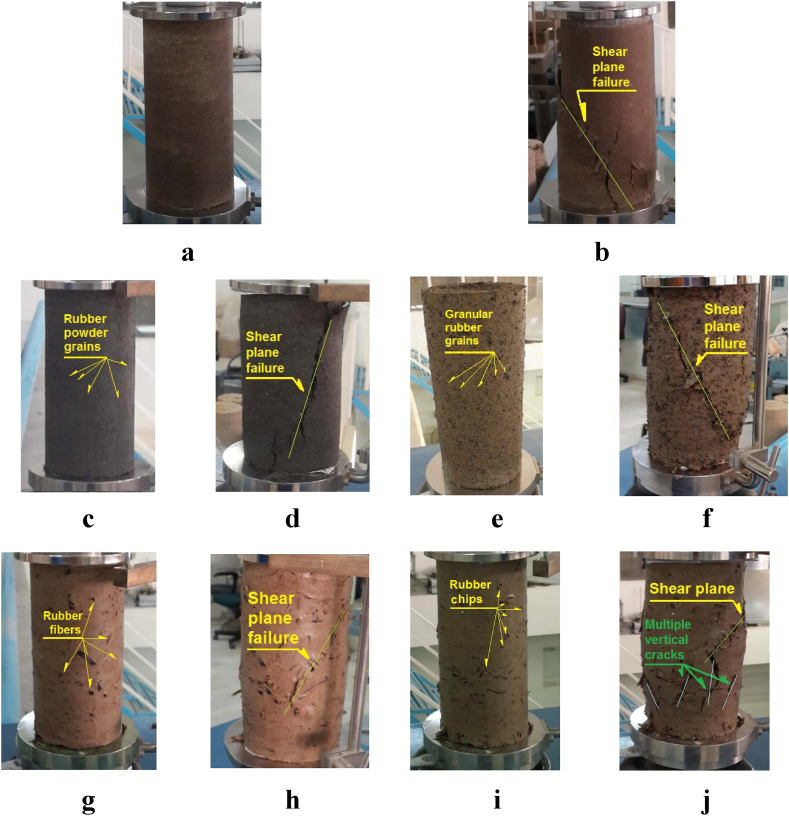
Initial and final state of the samples in UCS tests. (a) HPCS _100_RW_0_ (Initial sample). (b) HPCS _100_RW_0_ (After failure). (c) HPCS 76P24(Initial sample). (d) HPCS _76_P_24_ (After failure). (e) HPCS _76_G_24_ (Initial sample). (f) CHS_76_G_24_ (After failure). (g) HPCS_76_F_24_ (Initial sample). (h) HPCS _76_F_24_ (After failure). (i) HPCS _76_C_24_ (Initial sample). (j) HPCS _76_C_24_ (After failure).

**Fig. 12:**
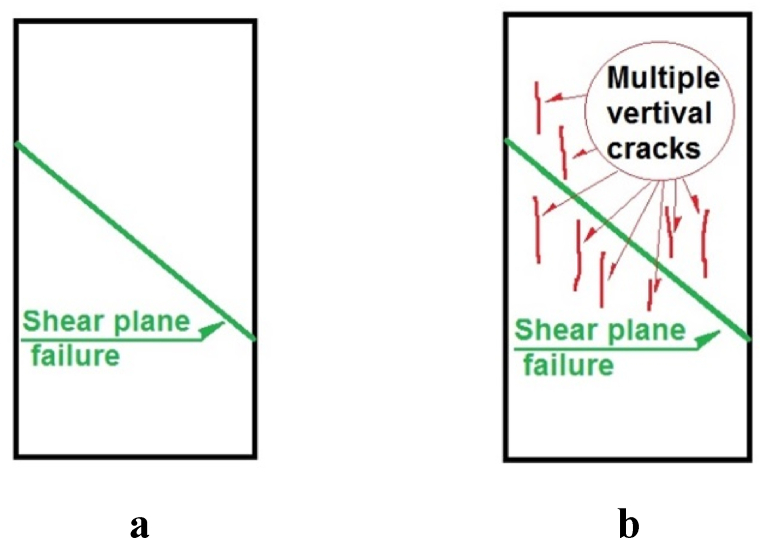
Type of failure modes observed HPCS-CRTW mixture in UCS tests. (a) Shear plane failure. (b) Combination of shear plane failure accompanied by multiple vertical cracks.

The type of failure observed in soil-CRTW mixtures is a crucial aspect worthy of investigation, as it is influenced by both the soil type and the CRTW characteristics. Fig. 11(a-j) illustrates the primary and secondary failure modes observed in the HPCS-CRTW specimens with varying CRTW content. The subsequent discussion delves into the overarching failure mechanisms exhibited by these specimens. As depicted in Fig. 12a, b, these specimens can be categorized into two main groups based on their failure modes: 1) shear plane failure and 2) a combination of shear plane failure accompanied by multiple vertical cracks. In the case of pure soil specimens, the predominant failure mode was the first type, characterized by a distinct and visible shear failure plane. This clay specimen exhibited substantial strength and displayed a well-defined failure line. Similarly, the specimens containing rubber powder also exhibited the same type of failure, namely shear plane failure.

The specimens containing granular rubber also displayed a distinct failure line and exhibited the same shear plane failure mode as the pure soil specimens. Similarly, the specimens with rubber fibers also featured a failure line akin to that observed in pure clay and the mixtures containing granular rubber. However, in addition to the failure line seen in other specimens, those containing rubber chips exhibited multiple vertical cracks. This observation suggests that the specimens with rubber chips are weaker compared to the other mixtures. These vertical cracks are a consequence of reduced cohesion and, consequently, reduced tensile strength in the mixture, which also has adverse effects on peak strength, shear modulus, and failure strain in these specimens.

Previous studies conducted by other researchers have reported varying behaviors in clay-CRTW mixtures depending on the type of soil and rubber used. In line with studies conducted by Sarvade and Shet (2012) and Akbarimehr et al. (2020) [49,52], adding rubber to clay results in a more ductile behavior. While this study focused on HPCS, which exhibits somewhat different plasticity properties, the results align with the findings of these earlier works.

## 4 Conclusions

This study delved into the behavior of HPCS mixtures with varying proportions of CRTW, encompassing granular rubber, rubber powder, rubber fiber, and rubber chips. Several critical parameters were scrutinized, including peak strength, post-peak softening behavior (PPSL), modulus of elasticity, stress-strain characteristics, result repeatability, and failure analysis. The principal findings can be summarized as follows:

• Repeatability of Test Results: The specimens containing granular rubber and rubber powder demonstrated the highest repeatability, with percentages of 94% and 95%, respectively. Slightly lower repeatability levels of 92% and 90% were observed for the specimens containing rubber fiber and rubber chips, respectively.
• Modulus of Elasticity: The specimens containing granular rubber exhibited the highest modulus of elasticity, while the lowest was recorded for those with rubber chips. A substantial drop in modulus was observed once the rubber content exceeded 6%, reaching values of 211 kPa and 161 kPa at 6% CRTW, respectively. Beyond 12% CRTW, further increases had a limited impact on this modulus. On average, mixtures containing granular CRTW displayed a 15% higher modulus of elasticity compared to those with other rubber shapes.
• Failure Strain: The study revealed a sharp reduction in PPSL upon adding CRTW to pure clay. Specimens with rubber fiber and granular rubber exhibited the highest failure strains, while those containing rubber chips showed the lowest. All specimens displayed significant increases in failure strain when the rubber content exceeded 6%. For instance, the mixture of HPCS with granular rubber achieved a failure strain of 5.2%, with HPCS-CRTW mixtures demonstrating approximately 25% higher shear strains on average compared to pure HPCS.
• Peak Strength Analysis and Predicting the UCS: Specimens containing granular rubber displayed the highest UCS, whereas those with rubber fiber and rubber chips exhibited lower strengths. Beyond 6% rubber content, a significant decrease in peak strength was observed. The results further indicated that mixtures with granular CRTW boasted an average 10% higher strength than those with powdered CRTW. For instance, the strength of the HPCS mixture with 6% granular rubber measured 310 kPa. Four equations have been provided to estimate the strength of HPCS mixtures with varying quantities of CRTW in different shapes.
• Failure Modes: Two primary failure modes were identified in the HPCS-CRTW specimens: shear plane failure, observed in pure soil specimens and those containing granular and fiber CRTW; and a combination of shear plane failure and multiple vertical cracks, observed in specimens containing rubber chips, which generally exhibited weaker behavior.

## Data availability statement

Data will be made available on request.

## Additional information

No additional information is available for this paper.

## CRediT authorship contribution statement

**Abolfazl Eslami:** Conceptualization, Methodology, Supervision, Writing – review & editing. **Davood Akbarimehr:** Conceptualization, Formal analysis, Investigation, Methodology, Software, Writing – original draft, Writing – review & editing. **Alireza Rahai:** Conceptualization, Methodology, Supervision, Writing – review & editing. **Moses Karakouzian:** Conceptualization, Methodology, Supervision, Writing – review & editing.

## Declaration of competing interest

The authors declare that they have no known competing financial interests or personal relationships that could have appeared to influence the work reported in this paper.
